# A novel framework of MOPSO-GDM in recognition of Alzheimer's EEG-based functional network

**DOI:** 10.3389/fnagi.2023.1160534

**Published:** 2023-06-29

**Authors:** Ruofan Wang, Haodong Wang, Lianshuan Shi, Chunxiao Han, Qiguang He, Yanqiu Che, Li Luo

**Affiliations:** ^1^School of Information Technology Engineering, Tianjin University of Technology and Education, Tianjin, China; ^2^Tianjin Key Laboratory of Information Sensing and Intelligent Control, School of Automation and Electrical Engineering, Tianjin University of Technology and Education, Tianjin, China; ^3^College of Agronomy, Sichuan Agricultural University, Chengdu, China

**Keywords:** Alzheimer's disease, EEG, complex network, multi-objective optimization, feature selection

## Abstract

**Background:**

Most patients with Alzheimer's disease (AD) have an insidious onset and frequently atypical clinical symptoms, which are considered a normal consequence of aging, making it difficult to diagnose AD medically. But then again, accurate diagnosis is critical to prevent degeneration and provide early treatment for AD patients.

**Objective:**

This study aims to establish a novel EEG-based classification framework with deep learning methods for AD recognition.

**Methods:**

First, considering the network interactions in different frequency bands (δ, θ, α, β, and γ), multiplex networks are reconstructed by the phase synchronization index (PSI) method, and fourteen topology features are extracted subsequently, forming a high-dimensional feature vector. However, in feature combination, not all features can provide effective information for recognition. Moreover, combining features by manual selection is time-consuming and laborious. Thus, a feature selection optimization algorithm called MOPSO-GDM was proposed by combining multi-objective particle swarm optimization (MOPSO) algorithm with Gaussian differential mutation (GDM) algorithm. In addition to considering the classification error rates of support vector machine, naive bayes, and discriminant analysis classifiers, our algorithm also considers distance measure as an optimization objective.

**Results:**

Finally, this method proposed achieves an excellent classification error rate of 0.0531 (5.31%) with the feature vector size of 8, by a ten-fold cross-validation strategy.

**Conclusion:**

These findings show that our framework can adaptively combine the best brain network features to explore network synchronization, functional interactions, and characterize brain functional abnormalities, which can improve the recognition efficiency of diseases. While improving the classification accuracy of application algorithms, we aim to expand our understanding of the brain function of patients with neurological disorders through the analysis of brain networks.

## 1. Introduction

Alzheimer's disease (AD) is the most common type of dementia, which is a neurodegenerative disease characterized by progressive cognitive decline, accompanied by a decrease in daily living ability and neuropsychiatric symptoms or behavioral changes (Zvěřová, 2019). The prevalence of AD increases rapidly at age 65. It currently affects ~55 million people worldwide and could increase twofold by 2050 (Niotis et al., 2022). But in fact, the disease often has a latent phase that is extremely difficult to detect. In recent years, the diagnosis and treatment of AD in its early stages has led to growing concern (Elmaleh et al., 2019; Zvěřová, 2019; Pais et al., 2020; Niotis et al., 2022). Clinically, the probable AD patients are normally evaluated with standardized neuropsychological tests such as the MMSE (Mini-Mental State Examination) (Tombaugh, 2004; Grassi et al., 2019; Qiu et al., 2019; Abbate et al., 2021). In addition, various studies have been proposed by numerous researchers to examine the predictive power of neurophysiological data respecting AD and other dementia symptoms (Salas-Gonzalez et al., 2011; Chaves et al., 2012; Swietlik and Bialowas, 2019; Mehmood et al., 2021).

Nevertheless, electroencephalography (EEG) has been widely used to track brain electrical activity generated by the cortical regions of the brain in Alzheimer's disease, due to its elevated temporal resolution, non-invasiveness, and relatively low cost compared to imaging tools (Dauwels et al., 2010; Wang et al., 2014). EEG represents the overall electrical activity of the brain in a waveform arising from myriad neuronal activities (Wang et al., 2015b). The more prevalent view is that the development of Alzheimer's disease is associated with a slowing of the EEG, a decrease in EEG complexity, and a disturbance of EEG synchronization (Wang et al., 2014, 2015b; Cao et al., 2015). Moreover, it is worth noting that in recent years, there has been rapid development in capturing the disconnection of Alzheimer's disease through functional network analysis (Joudaki et al., 2012; Cisler et al., 2014; Cai et al., 2018; Ismail and Karwowski, 2020; Gao et al., 2021).

Brain networks are considered a promising approach to capture the neuronal disconnection in Alzheimer's disease, and complex network theory has been applied to investigate functional and structural networks (Rubinov and Sporns, 2010; Vecchio et al., 2016; Sporns, 2022a,b). It has been found that healthy brains have high values in complex network analysis features such as small-worldness, hubness, and rich-clubs, while Alzheimer's disease brains have low values in these network features, indicating a disconnection of the brain in AD patients (Blinowska and Kaminski, 2013; Van den Heuvel and Sporns, 2013; Wang et al., 2014; Deng et al., 2015). Furthermore, substantia researches showed that network properties can capture the synergistic effect of abnormal paroxysmal firing of neurons more effectively than the characteristics of single channel signals (Wang et al., 2015b; Busche and Hyman, 2020; Gao et al., 2021). In addition, the combination of multiple features can help to display EEG abnormalities from various angles and improve the recognition efficiency (Mahato and Paul, 2020; Pei et al., 2020; Narayan, 2021; Wang et al., 2022). For instance, Narayan (2021) extracted and combined the features of AAR parameters, Barlow parameters, Hjorth parameters, etc. for the recognition of motor EEG signals. Wang et al. (2022) applied the second-order difference plot to extract geometric features for epileptic seizure detection of EEG. These results all lead to the consistent conclusion that a combination of features can improve classification accuracy compared to a single feature. Therefore, topological features based on complex network are considered to be extracted and further combined for AD brain network analysis in this paper.

However, as the number of features increases, the combination of extracted features may not consistently be able to accurately classify patterns in all classes (Ghosh et al., 2020). On the contrary, the accuracy of feature classification is related to (i) highly correlated features, which may result in redundancy in classification learning models or (ii) uncorrelated features, which may lead to pattern recognition failure (Khaire and Dhanalakshmi, 2022). Meanwhile, it is difficult to manually select features that are effective for classification tasks. To address this issue, feature selection has received widespread attention, as it can effectively remove irrelevant and redundant features and improve classification performance (Chandrashekar and Sahin, 2014).

In recent years, evolutionary computation techniques have received considerable attention for their global search capabilities. Commonly used evolutionary computation algorithms include particle swarm optimization (PSO) (Kennedy and Eberhart, 1995), ant colony optimization (ACO) (Ke et al., 2010), artificial bee colony (ABC) algorithms (Ren et al., 2017), and genetic algorithm (GA) (Holland, 1992). PSO was proposed by Kennedy and Eberhart (1995), due to their fast convergence speed and stable particle cooperation, it is widely applied in feature selection and highly efficient in applications with high-dimensional data (Samal et al., 2007; Tran et al., 2018; Chen et al., 2021; Wang et al., 2022). However, it is noted that PSO has a problem of premature convergence (Deng et al., 2017). Therefore, appropriately improving the PSO may help feature selection for high-dimensional data.

Additionally, it is crucial to determine appropriate evaluation metrics during training that can identify information that is effective for classification while minimizing the number of features. Most studies, in fact, use one objective or the weighted-sum method to combine two objectives for merging two objectives into a single objective. For instance, as one objective, the classification accuracy was used in PSO algorithm for depression detection (Akbari et al., 2021). For the weighted-sum method: (i) the number of features in training data and classification accuracy (Chuang et al., 2008), (ii) the fisher-discriminate-like criteria and training accuracy (Tran et al., 2017), were combined as one objective in PSO for feature selection. Significantly, in practice, a suitable weight parameter for the weighted-sum method is difficult to determine, especially for high-dimensional data, despite the use of trial and error tests. To address this issue, recent research has started to consider multi-objective optimization with three or more objectives, and it has been applied in many fields such as image processing (Gong et al., 2016), feature construction (Hammami et al., 2018) and cancer classification (Sharma and Rani, 2019). Moreover, compared with single-objective optimization, multi-objective optimization can consider the trade-off relationships between different objectives and generate a set of solutions rather than a single solution, which provides profound insight into the characteristics of the issue (Zhou et al., 2020).

In this study, brain network analysis theory is used to explore abnormal brain changes in AD patients. In order to construct it, phase synchronization index (PSI) is used to characterize the degree of correlation between EEG signal leads. Meanwhile, a total of 14 topological features are extracted to quantify brain structure. However, when all 14 features are used for classification analysis, the calculation becomes complex in the classification process and may be redundant due to some features, leading to the decline of recognition. In addition, the commonly used feature selection methods such as Recursive Feature Elimination (RFE) and Principal Component Analysis (PCA) only calculate between features, and ignore the feedback relationship between feature combination and classification or recognition. Thus, feature selection is implemented via the proposed multi-objective optimization algorithm: MOPSO-GDM. In addition to the classification error rate, a distance metric is considered an objective in MOPSO-GDM algorithm, which enables the algorithm to find the combination with the minimum number of features that characterize the structural changes of brain network in AD patients, and improve the operation efficiency of the algorithm.

The remainder of this paper is organized as follows. In Section 2, the AD dataset and data preprocessing are described. Section 3 elaborates on the proposed AD recognition methods, including PSI, topological feature extraction, MOPSO-GDM, and their applications in feature selection. Section 4 presents the analytical results, including brain network analysis, feature statistics analysis, feature classification analysis, and application analysis of MOPSO-GDM. Section 5 provides the discussion while Section 6 presents the conclusions.

## 2. Material

In this study, EEG signal data are mainly used for the analysis of abnormal brain structural changes in AD. Therefore, this section will detail the sources of EEG data from AD patients in this study and the preprocessing method.

### 2.1. Subjects

Thirty subjects were recruited in this study and divided into AD and normal control groups. Clinical neuroimaging and neurological examination comprised computed tomography (CT), structural MRI, cerebellar testing and cranial nerve examination, and Mini-Mental State Examination were executed to show the different clinical symptoms of the two groups, so to identify the potential network structure alterations in AD condition. All the AD patients were determined according to the international classification of diseases (ICD-10) of the world health organization and the diagnostic criteria of dementia in the Diagnostic and Statistical Manual of Psychiatric Disorders (DSM-IV). They were free of other neurological or psychological disorders, neurological active medications, or any other factors that may affect brain activity. The specific information of the subjects is shown in Table 1.

**Table 1:** Demographic and clinical characteristics of AD and healthy control subjects.

	**ADs**	**Healthy controls**
Subject (numbers)	15	15
Right-handed or left-handed	Right-handed	Right-handed
Age (years)	77.6 (±3.4SD)	72.2 (±1.9SD)
Gender (F/M)	8/7	9/6
Education (years)	>6	>6
MMSE (0–30)	21.3 (±5.8SD)	27.1 (±1.3SD)
CDR (0–3)	1.0 (±0.5SD)	-
GDS (0–15)	4.2 (±2.4SD)	-

MMSE, mini-mental scale examination; CDR, clinical dementia rating; GDS, global deterioration scale; SD, standard deviation.

### 2.2. EEG acquisition and preprocessing

**Figure 1:**
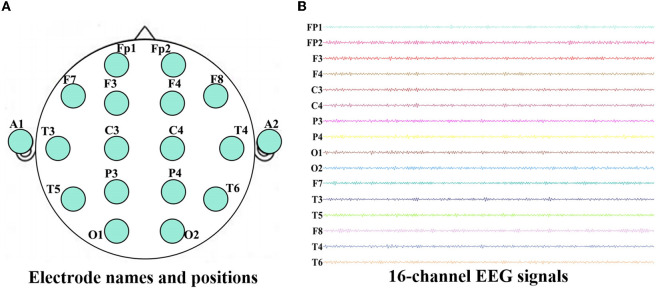
Electrode names and positions on the brain **(A)** and 16-channel EEG signals recorded for one AD patient **(B)**.

This experiment was collected in the First Ward of Neurology Department, Huanhu Hospital, Tianjin, China. During the experiment, all the subjects were seated upright and kept awake in a semi-dark, quiet room with electromagnetic shielding. Furthermore, they were told in advance to avoid any movement, such as body actions, eye movements and blinking. For each subject, EEGs were collected for ten min (the eyes were closed for the first five minutes, and the eyes were open for the next five minutes) using a 16-channel Symptom amplifier (Symtop Instrument, Beijing, China) with a sampling rate of 1024 Hz and bandpass filtered between 0–60 Hz. Sixteen Ag-AgCl scalp electrodes Fp1, Fp2, F3, F4, C3, C4, P3, P4, O1, O2, F7, F8, T3, T4, T5, T6 were set on the scalp according to the international 10–20 system, and the linked earlobe A1 and A2 are used as a reference, as shown in Figure 1A. In the process of data acquisition, all steps are completed and monitored by the experienced experimenter to ensure the correctness and effectiveness of the EEG recordings.

To avoid the influence of the machine starts, we take the data of the middle period (2–4 min) of the eyes closed state in the first 5 minutes as the analysis object. This paper uses AD original data from a total of 15 participants, with a sampling frequency of 1,024 Hz. Each participant was sampled for 2 minutes (120 seconds). The EEGs of all subjects were selected and segmented according to the length of 10 seconds (1,024 Hz–10 s = 10,240), increasing the total number of samples for AD data to 180. The raw EEG data recorded during the experiment are shown in Figure 1B. In order to remove the volume conduction effect, the selected EEG recordings are preprocessed according to the method mentioned in Wang et al. (2014, 2015b) and Cai et al. (2018). Then each channel of EEG recordings is decomposed into five sub-bands: delta (δ, 1–4 Hz), theta (θ, 4–8 Hz), alpha (α, 8–12Hz), beta (β, 12–30 Hz) and gamma (γ, 30–60 Hz) via the finite impulse response (FIR) filter. All procedures are implemented in a MATLAB R2021b (9.11.0.1769968) environment.

## 3. Method

The EEG signals of AD patients show the characteristics of slow rhythm, reduced complexity and synchronous disturbance. At the same time, functional network is an efficient research method to study brain functional changes. Therefore, the effective method of AD recognition through EEG features can perform signal detection faster and identify more accurately by machine learning methods compared with manually labeling AD EEG features. In this section, based on the “disconnection” nature of brain neurons in Alzheimer's disease, a brain functional network construction method based on Phase Synchronization Index (PSI) is proposed. The network topological characteristics are described according to graph theory, and the MOPSO-GDM algorithm is proposed to perform multi-objective optimization of the characteristics. The details are as follows.

### 3.1. Construction of brain functional network

Brain networks can be constructed to identify functional and structural alterations in the underlying brain networks of AD patients, where the network construction requires the determination of two main factors: nodes and edges. Thus, each brain electrode channel is taken as the node, and the function connection of the two channels is calculated as the edge by PSI. The PSI is one of the commonly used metrics to measure the connection strength of network functions, which can describe the relationship in the instantaneous phases between time series. Even if the amplitude of the time series is statistically independent, the instantaneous phase may be completely synchronized. To compute the PSI, the Hilbert transform is applied to extract the instantaneous phase of all raw EEG signals. Suppose *x*(*t*) is any one channel of EEG, and $\tilde{x}\left(t\right)$ is its Hilbert transform, which is defined by:


(1) $$\begin{array}{l} \tilde{x}\left(t\right)\text{=}\frac{1}{\pi }pv\overset{\infty }{\underset{-x}{\int }}\frac{x\left(\tau \right)}{t-\tau }d\tau \end{array}$$

where *pv* is the Cauchy principal value. Then both the instantaneous amplitude *A*(*t*) and the instantaneous phase φ(*t*) can be computed by:


(2) $$\begin{array}{l} \begin{array}{c} A\left(t\right)\text{=}\sqrt{{\left[x\left(t\right)\right]}^{2}+{\left[\tilde{x}\left(t\right)\right]}^{2}}\\ \varphi \left(t\right)\text{=}\arctan\frac{\tilde{x}\left(t\right)}{x\left(t\right)} \end{array} \end{array}$$

Then the phase difference between the EEG signals is defined as:


(3) $$\begin{array}{l} \Delta \varphi \left(f_{m},f_{n},t\right)\text{=}m\varphi \left(f_{m},t\right)-n\varphi \left(f_{n},t\right) \end{array}$$

where *f*_*m*_ and *f*_*n*_ are the center frequencies of the EEG signals. Besides, *m* and *n* are integers which should satisfy the inner product formula *m*·*f*_*n*_ = *n*·*f*_*m*_. Here, *m* = *n* = 1. So the PSI can be calculated by:


(4) $$PSI\left(f_{m},\;f_{n}\right)=\left|\langle e^{j-\left(\Delta \varphi \left(f_{m},\;f_{n},\;t\right)\right)}\rangle \right|$$

Seen from Equation (4), the value range of PSI is [0, 1]. 0 indicates that there is no coupling or coupling delay is zero, and 1 is full (non-zero delay) phase locking. The larger the PLI value, the higher the synchronization degree between the EEG signals. Furthermore, since zero-lag synchronization is removed from the analysis, PSI is relatively insensitive to the volume conduction effect. For above, in this study, PSIs between each two channels can be calculated to generate five 16 × 16 connectivity matrices, which shows the synchronization in five frequency bands when considering the symmetry. According to graph theory, the PSI matrix can be transformed into an unweighted binary adjacency matrix *A*_*ij*_ by applying a given threshold τ (τ is 0.3). In the representation of the functional network graph, only connections with PSI values greater than the threshold τ are realized, indicated by the corresponding entry *a*_*ij*_ in the matrix being 1, otherwise it is 0.

### 3.2. Extraction of brain network features

Network measures are often represented in multiple ways with the aim of characterizing local specialization and global integration of brain network. In this section, measures are described that variously detect aspects of functional integration and segregation, quantify the importance of individual brain regions, characterize patterns of local anatomical circuitry, and test the resilience of networks to insult. The most commonly used features of brain functional network are listed in Supplementary material: Degree (DG), Node Betweenness (NB), Clustering Coefficient (CC), Shortest Path Length (SPL), Edge Betweenness (EB), Global Efficiency (GE), Local Efficiency (LE), Transitivity (TT), Assortative Coefficient (AC), Small-Wordless (SW), Modularity (MD), Motif z-score (MZ), Hierarchical Coefficient (HC), and Graph Index Complexity (GIC).

### 3.3. Multiple-objective particle swarm optimization with Gaussian difference mutation (MOPSO-GDM)

In general, combining features improves classification performance. However, in addition to the features being undeterminable, the number of features in the combination is equally undeterminable. It is worth noting that when the 14 brain network features are arranged and combined, it will produce 2^14^ feature combinations. Meanwhile, combining features manually or programmed by a computer and feeding them into a classifier will consume a lot of time. Therefore, in this study, the use of intelligent algorithms to find feature combinations has become the core idea of AD recognition framework. Different from other feature selection methods, the proposed feature selection method uses the classification result of feature combination as the feedback result to the intelligent algorithm, and uses it to search the solution space of feature combination purposefully.

**Figure 2:**
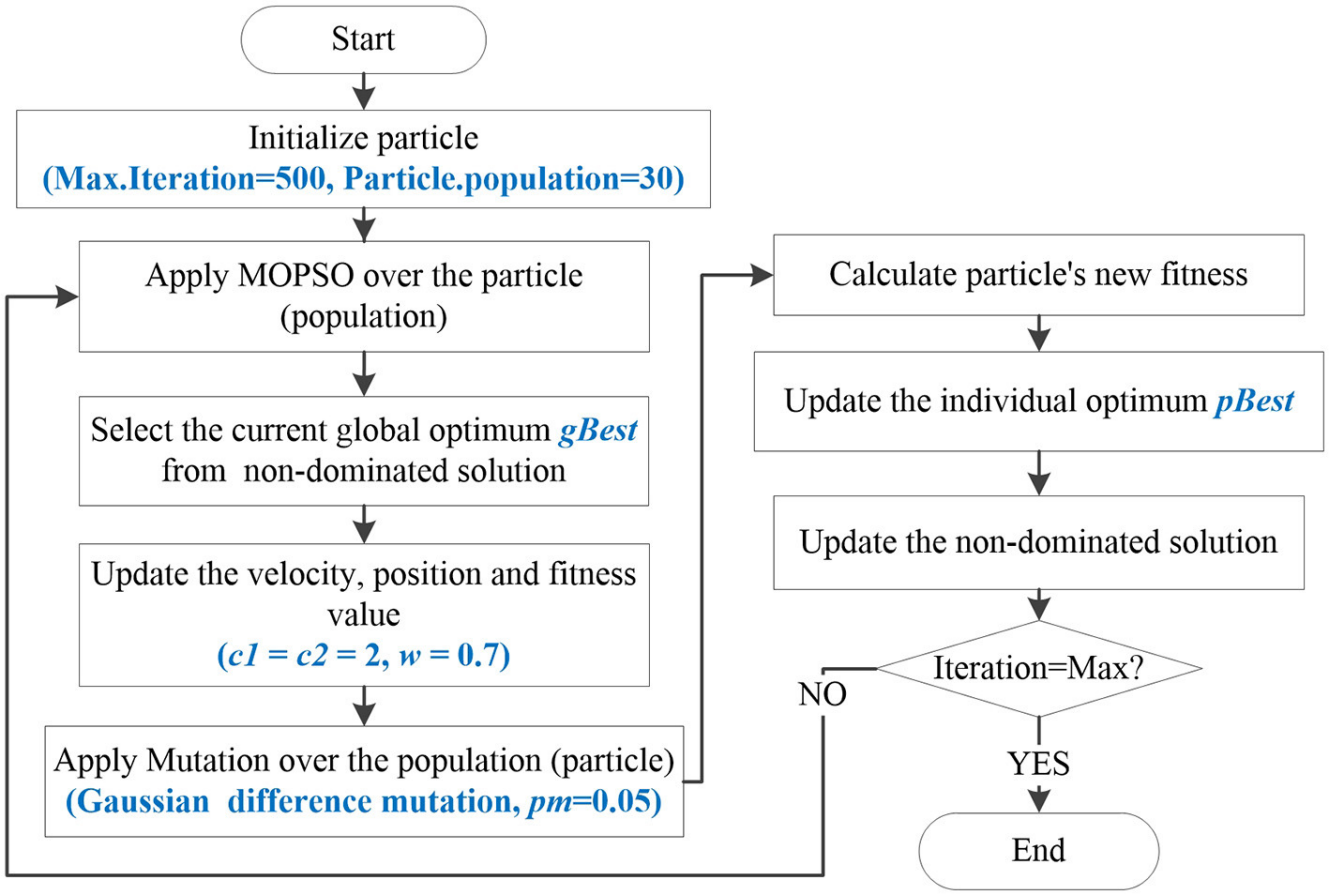
The flow-chart of MOPSO-GDM.

Furthermore, due to the large number of brain network feature combinations, the solution space becomes larger. For this issue, a mutation operation is proposed to increase the search space of the algorithm. Then, an improved Multiple- objective optimization algorithm, MOPSO-GDM is proposed, which combines MOPSO with the Gaussian difference mutation (GDM). In each iteration, particles are first optimized by MOPSO with the learning factor *c*_1_, *c*_2_ set to 2 and the weight coefficient *w* set to 0.7. Then, send all optimized particles to the mutant of Gaussian differential mutation (GDM) to prevent some particles from being trapped in local optimization, with a mutation rate set to 0.1. Among them, GDM is performed between random individuals in the particles, the optimal and the current positions of the particles. Since GDM can generate larger perturbations near the current mutation, it is easier for the algorithm to jump out of the local extreme value. The mathematical expression is as follows:


(5) $$\begin{array}{l} L\left(t+1\right)=p_{1}\times f_{1}\times \left(L^{*}-L\left(t\right)\right)+p_{2}\times f_{2}\times \left(L_{rand}-L\left(t\right)\right) \end{array}$$

where *p*_1_, *p*_2_ are weight coefficients with the value of 0.5. *f*_1_, *f*_2_ are the coefficients of Gaussian distribution function, which take the random number function of Gaussian distribution with the mean of 0 and the variance of 1. L^*^ is the optimal position of particle, namely *pbest*. *L*_*rand*_ is the position vector of particle randomly selected. *L*_*t*_ is the current position of the particle in iteration *t*. The flow-chart of the proposed algorithm is shown in Figure 2.

### 3.4. Application of MOPSO-GDM in feature selection

The MOPSO-GDM is applied as follow: first, sort the features in the following order: DG, NB, CC, SPL, EB, GE, LE, TT, AC, SW, MD, MZ, HC, and GIC. Randomly combine these features to represent the algorithm particles. Each particle consists of 0-1 loci with a length of 14/70 bits (14 bits for single-band, and 70 bits for five-bands), where the number 1 indicates that the feature is selected as an input to the classifier, and vice versa. For example, suppose that the feature combination in the single-band setup is represented by the particle [01000000010001 (δ)]; accordingly, the features NB (2nd), SW (10th), and GIC (14th) are selected for combination. Second, in order to find the classification stability of feature combination, the classification error rate of support vector machine (SVM), naive bayes (NB), and discriminant analysis (DA) classifiers are chosen as the objective value (*F*_1_, *F*_2_, and *F*_3_) of the algorithm. Moreover, in the feature selection, the number of selected features and classification error rate are considered as two conflicting objectives, while in this work, distance measurement is used to evaluate the classification performance. Therefore, fisher's discriminant distance measure introduced in Al-Sahaf et al. (2015) constitutes the fourth objective of our problem. By using distance measurement, the distance between samples of the same class can be reduced, while the distance between samples of different classes can be increased. The equation is as follows:


(6) $$\begin{array}{l} F_{4}=\frac{1}{1+\exp^{-\left(D_{w}-D_{b}\right)}} \end{array}$$

where the sigmoid function ensures that the value of *F*_4_ ranges from 0 to 1. *D*_*b*_ and *D*_*w*_ represent the average distance of each sample to the farthest sample within a different class and the average distance of each sample to the closest sample within the same class, respectively. *D*_*b*_ and *D*_*w*_ are calculated using Equations (7) and (8), respectively.


(7) $$\begin{array}{l} D_{b}=\frac{1}{\left|T\right|}\overset{\left|T\right|}{\underset{i=1}{\sum }}\underset{\left\{j|j\neq i,class\left(S_{i}\right)\neq class\left(S_{j}\right)\right\}}{\min}Dis\left(S_{i},S_{j}\right) \end{array}$$

(8) $$\begin{array}{l} D_{w}=\frac{1}{\left|T\right|}\overset{\left|T\right|}{\underset{i=1}{\sum }}\underset{\left\{j|j\neq i,class\left(S_{i}\right)=class\left(S_{j}\right)\right\}}{\max}Dis\left(S_{i},S_{j}\right) \end{array}$$

where |*T*| represents the total number of samples in the training set and *Dis*(*S*_*i*_, *S*_*j*_) denotes the Euclidean distance between the two samples *S*_*i*_ and *S*_*j*_.

**Figure 3:**
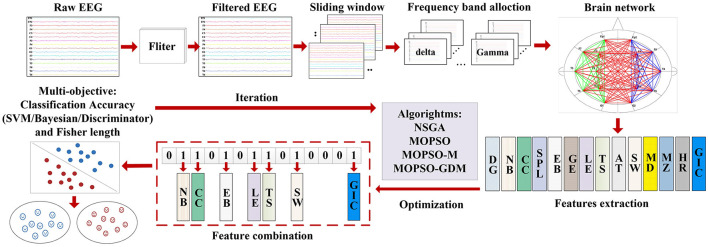
Intelligent process for AD recognition.

In addition, the model applies 10-fold cross-validation, with a ratio of 9:1 between training data and test data. Due to the real numbers in the algorithm, the real numbers of the particles need to be converted to 0-1 through threshold before being input into the classifier. The intelligent detection process is shown in Figure 3.

## 4. Results

In this section, 14 features extracted from five frequency bands are statistically analyzed (One-way ANOVA), and the features were sent to the SVM classifier for classification analysis. In order to improve the recognition efficiency of AD, the features are combined, and multi-objective optimization algorithms: NSGA, MOSPO, MOPSO-M, and MOPSO-GDM are used for feature selection to find the optimal feature combination to characterize the abnormal changes in AD brain. The results are analyzed as follows.

### 4.1. Brain network analysis

**Figure 4:**
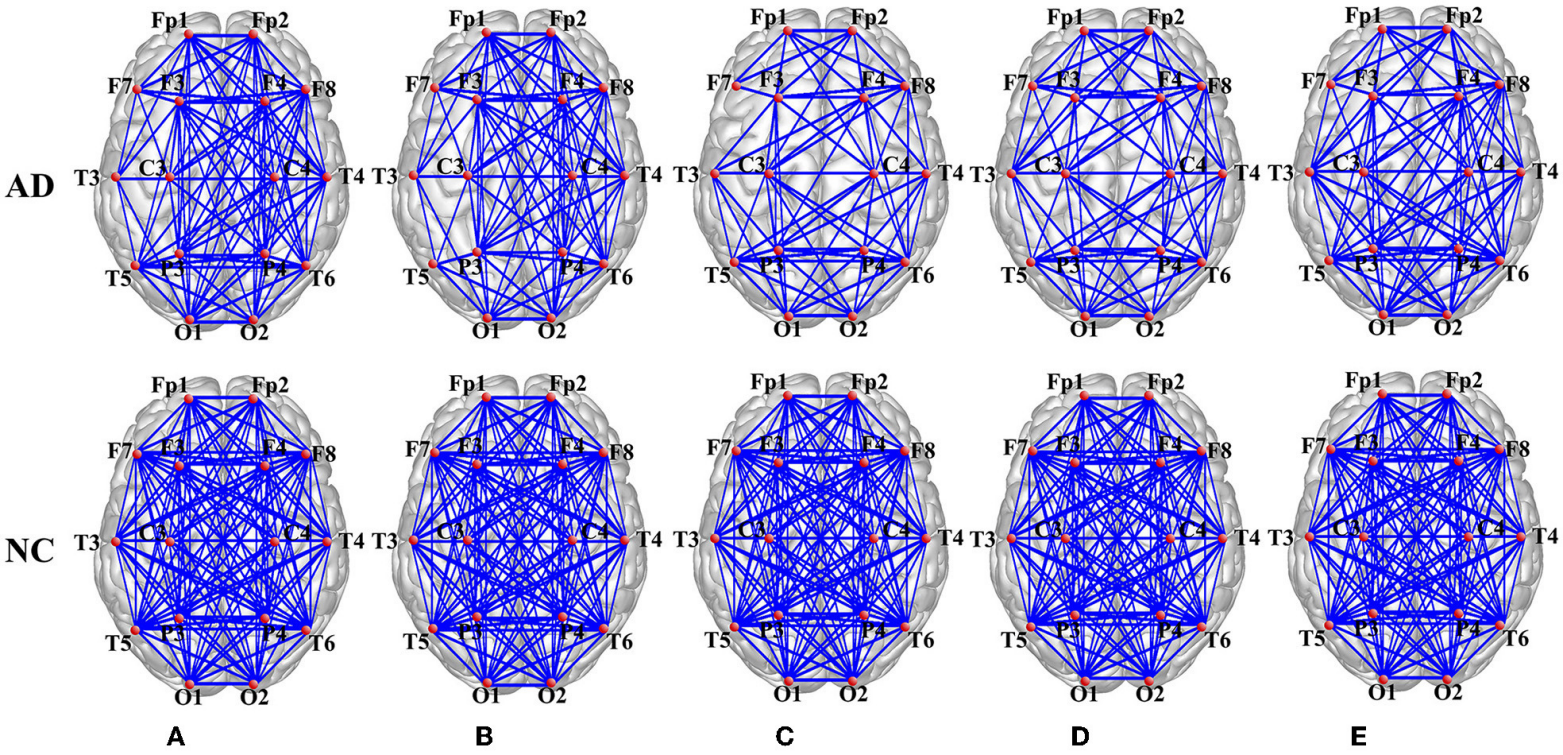
Brain network visualization in the **(A)** delta (δ), **(B)** theta (θ), **(C)** alpha (α), **(D)** beta (β), and **(E)** gamma (γ) frequency band.

Through the PSI method, the PSI matrix (16–16 connectivity matrices) is obtained to investigate the synchronization dynamics of AD patients and the normal controls (NC). After thresholding, PSI matrix transferred to adjacency matrix, which can be visualized through functional connectivity of AD and the normal control group in the δ, θ, α, β, and γ frequency band, as shown in Figure 4.

It is clear that the average network connectivity of AD patients decreases in each frequency band compared to the normal control group. Especially in the α, β, and γ frequency bands, there are significant differences between the AD and normal groups, while the difference in the δ and θ frequency bands are reduced. Therefore, the conclusion can be drawn that the functional network connectivity strength changes significantly in the AD brain and that the difference between AD and control is more pronounced in the high frequency band and weaker in the low frequency band.

### 4.2. Extraction and analysis of network features

**Figure 5:**
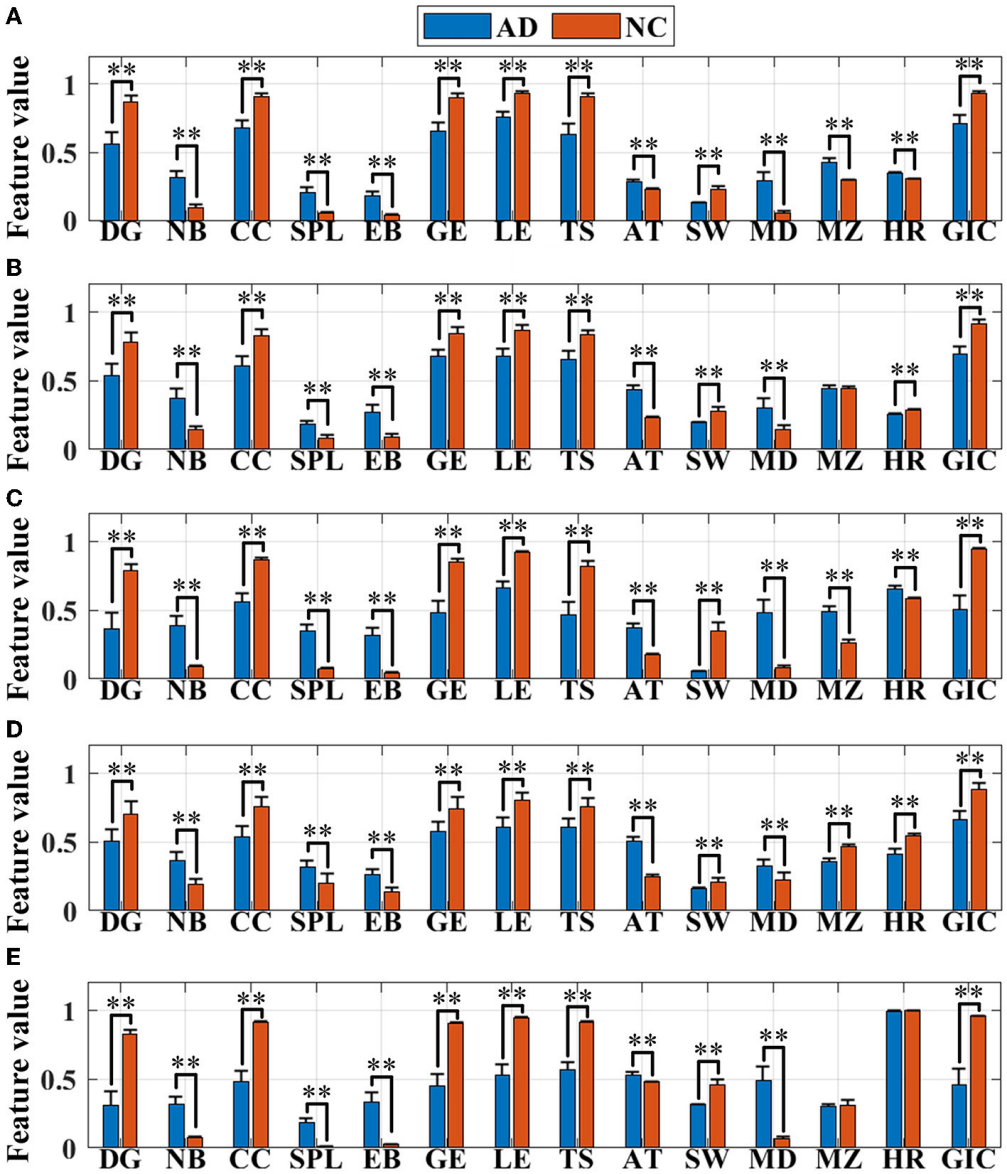
Feature (normalized in the range of [0,1]) visualization in the **(A)** delta (δ), **(B)** theta (θ), **(C)** alpha (α), **(D)** beta (β), and **(E)** gamma (γ) frequency bands. Asterisks represent significant differences between the two groups (**P < 0.01).

The following 14 network topological features of AD and normal brains are extracted for further quantitative analysis, which are shown as histograms in Figure 5. One way ANOVA is implemented to study whether there are significant differences between the two groups, “**” represents there exists obvious group difference with P < 0.01, as shown in **Figure 7** and Supplementary Table S1. There are significant differences between the features of AD brain network and the normal controls in the five frequency bands, except for the features of MZ in the θ frequency band, MZ and HR in the γ frequency band.

It can be seen that the remaining 12 features in both groups, with the exception of MZ and HR, show consistent results in all five frequency bands. First, the values of DG, CC, GE, LE, TS, SW, and GIC in AD group are lower than those in the normal control group, indicating that compared to the normal brain, the network connectivity in the AD brain is sparser, with the worse local aggregation, the lower global efficiency of information transmission, the weaker abilities of information parallel processing and local information transmission, and the simpler brain functional structure, which could effectively explain the disconnection characteristics of the AD brain. The increase in the values of NB, SPL, EB, AT, and MD in the AD group suggests that the AD brain network is altered to be more susceptible to abnormalities, with longer information transmission paths, worse stability of network structure, lower efficiency of information transmission and interaction, slower speed, and higher degree of polarization but a looser topology associated with cognitive dysfunction.

Moreover, for module properties, there are significant differences in MZ in the δ, α, and β frequency bands. The MZ value of AD group is higher than that of the control group in the δ and α frequency bands, and the MD shows the same trend, manifesting that although the connectivity of AD brain network is greatly sparser than that of the normal brain, both the modularization degree and the importance of modules of AD network are higher in the two frequency bands. While in the β frequency band, the modularization degree of AD network is raised, but the importance of modules reduces. In addition, the hierarchy of brain network in the AD group reduces in the δ and α frequency band, reflecting in the significant reduction of HR value. Thus, for the ANOVA results, these features can be considered for the classification analysis.

### 4.3. Classification analysis of features

The average classification results are shown in Table 2, where cross-validation is performed with a 9:1 ratio of training and test set (training = 360 × 90%, test = 360 × 10%). For all features, the α and γ bands show the best classification effect, for which the lowest classification error rate reach 0.1487 (α) and 0.1409 (γ) in SPL, followed by the β band, for which the lowest error rate is 0.1754 in AT, whereas the δ and θ bands have the poor discrimination, with the lowest classification error rate of 0.2272 in MZ and 0.2485 in AT, respectively. Among the ten features, SPL perform best in the α and γ bands.

**Table 2:** The classification results of single features (DG-GIC) in the δ, θ, α, β, and γ frequency bands.

**Band**	**DG**	**NB**	**CC**	**SPL**	**EB**	**GE**	**LE**	**TS**	**AT**	**SW**	**MD**	**MZ**	**HR**	**GIC**
δ	0.2455	0.2567	0.2534	0.2647	0.2552	0.2544	0.2641	0.2566	0.2492	0.2959	0.2539	0.2272	0.4295	0.2876
θ	0.2569	0.2609	0.2752	0.258	0.2694	0.2553	0.2694	0.3258	0.2485	0.3735	0.3713	0.4332	0.4408	0.2638
α	0.1627	0.1657	0.2028	0.1487	0.1794	0.1743	0.1876	0.2434	0.166	0.1777	0.2208	0.2109	0.3512	0.157
β	0.2097	0.2753	0.2178	0.2217	0.2683	0.2012	0.2106	0.2332	0.1754	0.3321	0.3037	0.2774	0.3239	0.2669
γ	0.1505	0.2232	0.1469	0.1409	0.1692	0.1495	0.142	0.2392	0.2915	0.2139	0.2333	0.3202	0.2405	0.145

### 4.4. Application results of NSGA, MOPSO, MOPSO-M, and MOPSO-GDM

In this section, in order to improve the classification error rate in each frequency band, multiple features are combined, and multi-objective optimization optimization algorithms (NSGA, MOPSO, MOPSO-M, and MOPSO-GDM) are applied to determine the optimal feature combination. In particular, the mutation operation added in MOPSO-M is consistent with the mutation in NSGA.

#### 4.4.1. Simulation test

**Figure 6:**
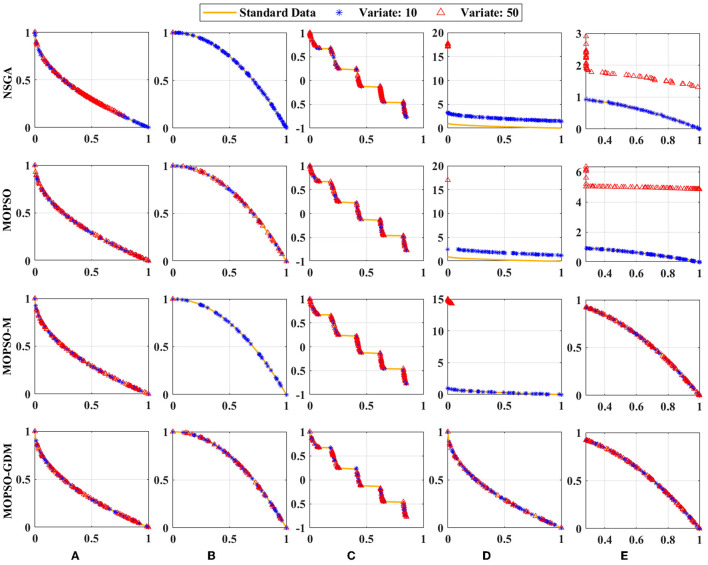
Simulation test results of the four algorithms with the **(A)** ZDT1, **(B)** ZDT2, **(C)** ZDT3, **(D)** ZDT4, and **(E)** ZDT6 functions. Standard data: yellow line; Variate 10: blue line; Variate 50: red line.

The four algorithms are used together with the Zitzler-Deb-Thiele ((ZDT1-ZDT4 and ZDT6) functions to verify their effectiveness, which are shown in Figure 6. These test functions have different properties, where ZDT1 has a convex Pareto front, ZDT2 has a non-convex Pareto front, ZDT3 has multiple disconnected convex Pareto fronts, ZDT4 has many local optima, the global Pareto front is non-convex, and ZDT6 has a non-convex Pareto front with a thin density toward the Pareto front (Li et al., 2010). Since ZDT5 is a Boolean function, it is not used in this paper. In, the particle and iteration numbers were set to 30 and 50, and the experiments were performed 20 times to avoid chance. It is interesting to note that all four algorithms can achieve the optimal values in ZDT1 and ZDT2 with the variate is 10 or 50. When variate is 10, the optimal values can be found in all but NSGA and MOPSO in ZDT1. Nevertheless, when variate is 50, ZDT1, ZDT4 and ZDT6 in NSGA could not find the optimal value, and the same is true for ZDT4 and ZDT6 in MOPSO, ZDT2 and ZDT4 in MOPSO-M. It is noted that MOPSO-GDM can find optimal values in all test functions: ZDT1-ZDT4 and ZDT6, suggesting that MOPSO-GDM can effectively avoid getting trapped in local optima and find optimal results in higher dimensions and more complex problems.

#### 4.4.2. Application analysis of AD recognition

**Figure 7:**
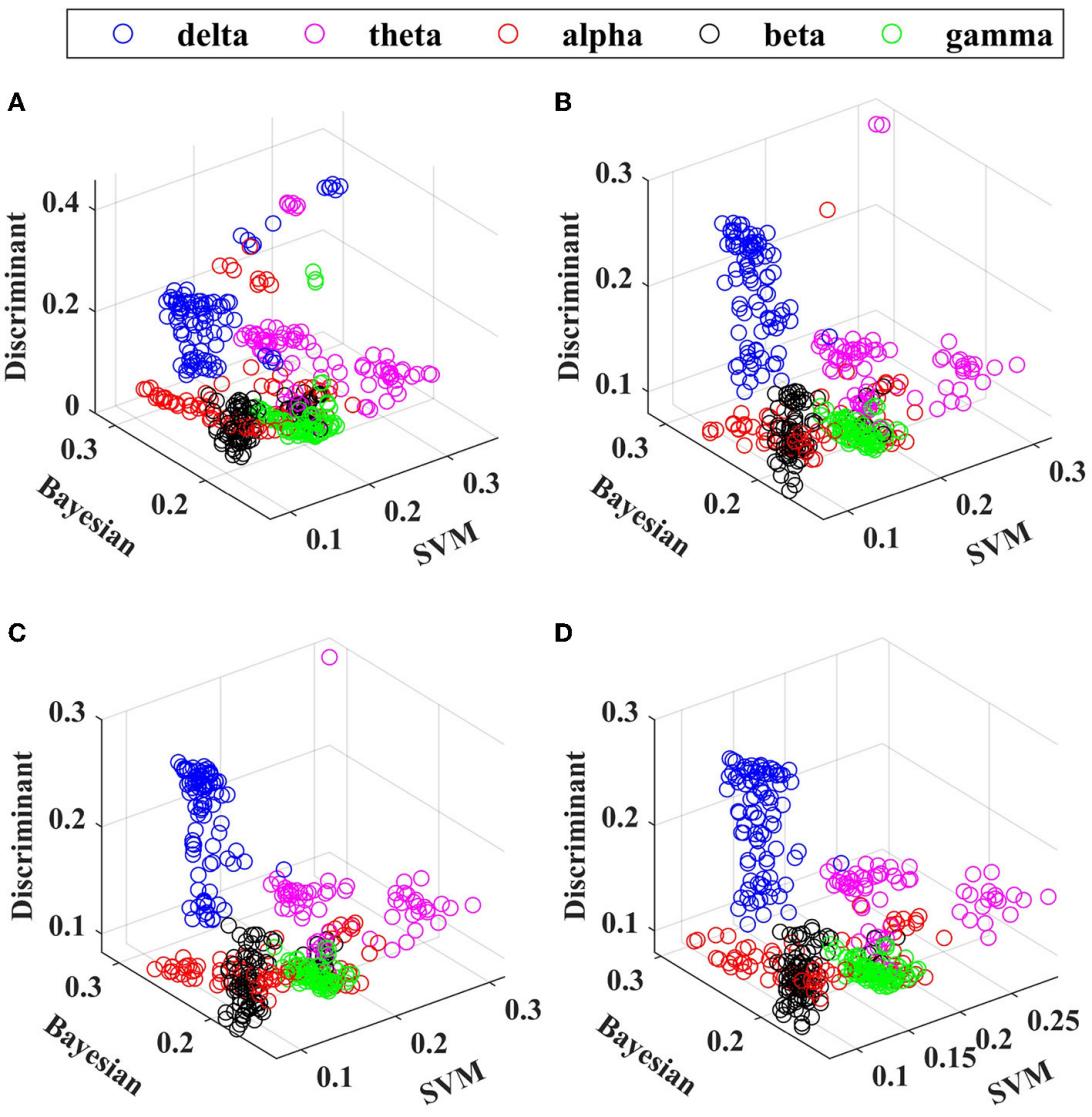
The scatter plot: non-dominated solution of **(A)** NSGA, **(B)** MOPSO, **(C)** MOPSO-M, and **(D)** MOPSO-GDM in the δ (blue point), θ (purple point), α (red point), β (black point), and γ (green point) frequency bands.

The features are combined in each frequency band, and NSGA, MOPSO, MOPSO-M, and MOPSO-GDM are applied to determine the optimal feature combination. A total of 20 experimental trials are conducted with each algorithm, and the non-dominated solution set is displayed in the form of scatter plot, as shown in Figure 7: three coordinates are SVM, NB, and DA classification error rates, respectively, where the value of error rates are shown in Table 3. It can be seen that feature combinations reach lower error rates than single feature (Table 2) with SVM. In addition, the lowest error rate emerges in β frequency band, particularly in MOPSO-GDM, i.e., 0.1319 (SVM: 0.0933, NB: 0.1731, and DA:0.1292). Then followed by α and γ frequency bands with error rates of 0.1445 in MOPSO (SVM: 0.1237, NB: 0.1856, and DA: 0.1242) and 0.1326 in MOPSO-GDM (SVM: 0.1248, NB: 0.1404, and DA: 0.1387), respectively. Moreover, error rates of δ frequency band is 0.1978 in MOPSO-M, θ band is 0.1884 in MOPSO-GDM, which are higher than other bands. It can be concluded that α, β, and γ frequency bands can get better classification performance by the way of feature combination, which is consistent with the results in Table 2.

**Table 3:** The SVM, NB, DA, and their average (AVG) classification error rates of non-dominated solution of NSGA, MOPSO, MOPSO-M, and MOPSO-GDM in the δ, θ, α, β, and γ frequency bands.

	**NSGA**				**MOPSO**				**MOPSO-M**				**MOPSO-GDM**			
	**SVM**	**NB**	**DA**	**AVG**	**SVM**	**NB**	**DA**	**AVG**	**SVM**	**NB**	**DA**	**AVG**	**SVM**	**NB**	**DA**	**AVG**
δ	0.1646	0.2672	0.2147	0.2155	0.1345	0.2652	0.207	0.2022	0.1308	0.2647	0.2174	0.1978	0.1339	0.2646	0.209	0.2025
θ	0.2361	0.2148	0.1501	0.2003	0.2219	0.2052	0.14	0.189	0.2229	0.2052	0.1383	0.2141	0.2242	0.2067	0.1344	0.1884
α	0.1411	0.2124	0.1437	0.1657	0.1237	0.1856	0.1242	0.1445	0.1219	0.1877	0.1204	0.1548	0.1249	0.1873	0.1232	0.1451
β	0.1148	0.1718	0.1288	0.1385	0.0954	0.1742	0.1333	0.1343	0.0996	0.1721	0.1271	0.1358	0.0933	0.1731	0.1292	0.1319
γ	0.1311	0.1442	0.1576	0.1443	0.1236	0.1407	0.1404	0.1349	0.1251	0.1401	0.1396	0.1349	0.1248	0.1404	0.1387	0.1326

**Figure 8:**
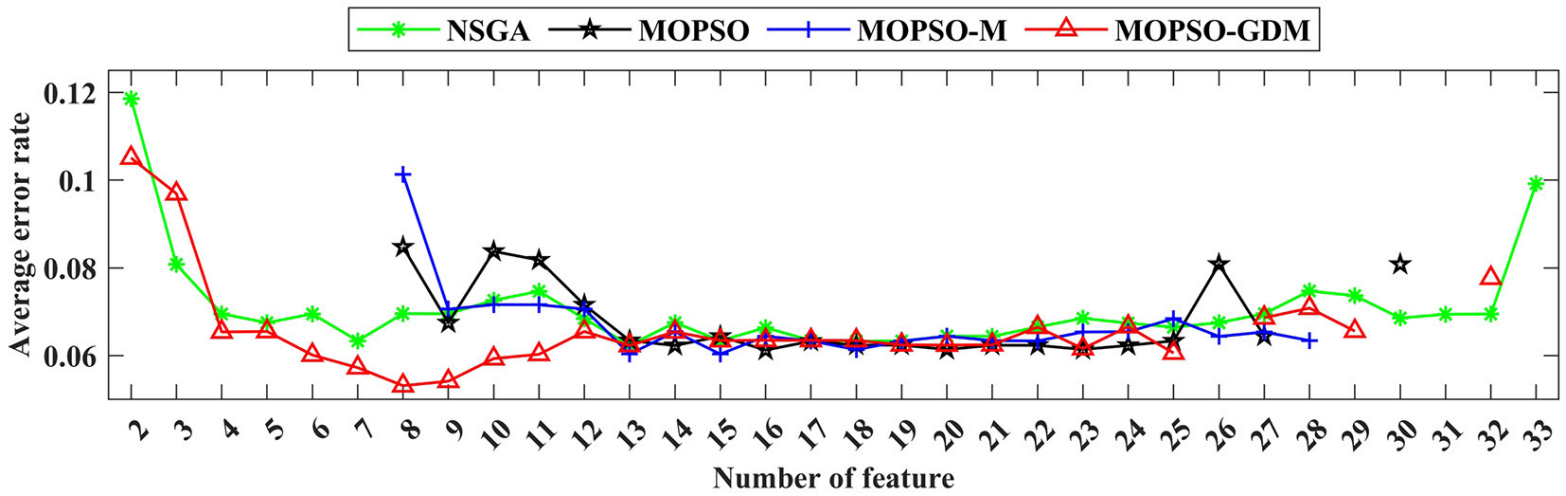
The variation trend of the minimum average classification error rate in the feature combination optimized by NSGA (green line), MOPSO (black line), MOPSO-M (blue line), and MOPSO-GDM (red line).

To further investigate the influence of frequency band combination to improve the recognition precision, the brain network features of five bands are combined and optimized by algorithms, where the number of particle variable is increased to 70 (14 features × 5 frequency bands), and the trend of minimum average error rate with the number of feature increased shows in Figure 8, the detail data show in Tables 4, 5. First, it can be found that the average error rates of five frequency bands combination of four algorithms were 0.0876, 0.0808, 0.0787, and 0.0769, lower than single frequency band (Table 3: γ frequency band), where MOPSO-GDM is superior to the other three algorithms. Second, the dimension of solution space of NSGA and MOPSO-GDM is the widest, which range from 2-33 and 2-32, respectively. It is followed by MOPSO and MOPSO-M, ranging from 8-30 and 8-28, respectively. Furthermore, the average error rate of the non-dominated solution optimized by MOPSO-GDM is the lowest, which is 0.0769. In addition, the min average error rate emerges in MOPSO-GDM with feature number of 8, is 0.0531 and better than other three algorithms, suggesting that MOPSO-GDM could find the optimal feature combination in multi-band combination, which is in accordance with the conclusion of Section 4.4.1 that MOPSO-GDM can find the optimal result in higher dimensions and more complex problems. As can be seen from the above, that combination of features between frequency bands may improve the efficiency of AD recognition.

**Table 4:** The SVM, NB, DA, and their average (AVG) classification error rates of non-dominated solution of NSGA, MOPSO, MOPSO-M, and MOPSO-GDM in five frequency bands combination.

	**The classification error rate of non-dominated solution**						
	**SVM**		**NB**		**DA**		**AVG error rate of 3 classifiers**
	**AVG**	**Var**	**AVG**	**Var**	**AVG**	**Var**
NSGA	0.0407	0.00091	0.1449	0.00433	0.0771	0.00262	0.0876
		(4.51e-05-0.0067)		(0.00051-0.0101)		(9.78e-08-0.0055)	
MOPSO	0.0151	0.00018	0.1534	0.0045	0.074	0.00348	0.0808
		(4.69e-05-0.0012)		(0.00157-0.0076)		(0.00016-0.0586)	
MOPSO-M	0.0108	0.00019	0.1481	0.00419	0.0772	0.00359	0.0787
		(4.68e-05-0.0015)		(0.00163-0.0097)		(0.00018-0.0586)	
MOPSO-GDM	0.0121	0.00022	0.143	0.00396	0.0755	0.00255	0.0769
		(4.69e-05-0.0044)		(0.0019-0.0093)		(0.00016-0.0089)	

**Table 5:** The minimum average (AVG) classification error rate and feature number of non-dominated solution of NSGA, MOPSO, MOPSO-M, and MOPSO-GDM in five frequency bands combination.

	**The non-dominated solution**	
	**AVG error rate (Min)**	**Number of features**
NSGA	0.0623	13
MOPSO	0.0612	16
MOPSO-M	0.0603	15
MOPSO-GDM	0.0531	8

## 5. Discussion

It is known that EEG features undergo changes in physiological and pathological aging (especially due to neurodegeneration), and the distribution of spectral power gradually changes (Vecchio et al., 2020, 2022), which has been shown in past studies that (in awake, resting conditions) a pronounced amplitude decrease of α band (Wang et al., 2015a,b; Elmaleh et al., 2019) and a global “slowing” of the background EEG rhythms, with the increase of δ and θ frequencies, the appearance of background EEG rhythms shows an overall diffusive phenomenon. This is in agreement with the findings in Figure 4 and Table 2. In addition, Gamma oscillations are prominent in multiple brain regions, including the hippocampus, and are believed to play a role in attentional selection and memory operations (Zeisel et al., 2015; Fernández-Ruiz et al., 2021; Benussi et al., 2022). In the present study, the γ band is studied and found to exhibit the same "slowing" property as the α band, and showed excellent classification performance with the average error rate of 0.1326, which is better than α band.

In addition, Figure 5 shows the group differences of global functional networks in degree, clustering coefficient, global efficiency, local efficiency and other topological parameters in five frequency bands. By these network measures, it can further investigate the changes of brain network “disconnection” in AD patients. Notably, this has been reported in previous papers, such as the average path lengths, clustering coefficients, global efficiency, local efficiency (Wang et al., 2014; Yu et al., 2020), where these features also appeared in the consistent results of this paper, that is, the value decreased in the AD brain network. Meanwhile, small-worldness (Wang et al., 2014), graph index complexity and modularity (Yu et al., 2020) are consistent with the results of this paper. But interestingly, the shortest path length decreased in the study by Yu et al. (2020), but increased in this study (Figure 5). Considering that Yu et al. used the single-channel network construction method: weighted visibility graph, which is different from the full-channel network construction by PSI. Thus, from the studies of La Foresta et al. (2019) and Duan et al. (2020), it can be found that shortest path length increased in AD full-brain network. Moreover, from various points of view of the network, features such as transitivity, betweenness of nodes and edges, assortative and hierarchical coefficients have been considered to study the topological differences in connectivity between two groups. All features indicate that the local and global functional networks in the AD group are significantly reduced, especially in the α, β, and γ frequency bands, which may lead to functional loss.

In this study, to overcome the lack of slow convergence, premature convergence, and tendency to get trapped in local optimal solutions in MOPSO, a novel modified method, MOPSO-GDM, is proposed and introduced to select the optimal feature combination. Owing to the combination of MOPSO and GDM, MOPSO-GDM achieves best performance in feature selection compared with NSGA, MOPSO and another improved PSO: MOPSO-M algorithms, especially in high-dimensional space optimization (Figure 7). From past studies, hybrid algorithms are commonly used as improved algorithms, such as GA-PSO (Gupta et al., 2019; Liu et al., 2020), BAPSO (Almadhor et al., 2021), PSO-GWO (Dahmani and Yebdri, 2020), and hybrid multi-objective optimization algorithms: NSGA-MOPSO (Shuaipeng et al., 2017; Xie et al., 2022). Nevertheless, most hybrid algorithms directly combined the whole of two algorithms, without considering the complexity of the algorithm (Wang et al., 2022). Therefore, in this paper, superfluous operators are eliminated and only mutation is added to simplify the algorithm flow. In addition, the categories of feature combinations searched by the algorithm during optimization are counted, as shown in Table 6. It is noted that MOPSO-GDM can seek additional feature combinations in 20 trials, that is, the search space is larger and the population is more diverse, thus better avoiding MOPSO from getting stuck in local optima than MOPSO-M.

**Table 6:** The number of feature combination category by searching in the optimization process of NSGA, MOPSO, MOPSO-M, and MOPSO-GDM.

	**The number of feature combination category (20 trials)**		
	**MIN**	**MAX**	**AVG**
NSGA	14787	16809	15233.4
MOPSO	8840	14028	12762.9
MOPSO-M	14944	15274	15041.45
MOPSO-GDM	16286	16560	16405.3

In addition, to verify the effectiveness of the algorithm results, we re-evaluate the non-dominated solution sets of the four classes of algorithms using the leave-one-out validation, and the results are shown in Table 7. First, it can be seen that the classification error rates using leave-one-out validation are also consistent with those shown in Table 4, among which the MOPSO-GDM algorithm still performs the best. Meanwhile, the optimal feature combination of the four algorithms in Table 5 are verified by leave-one-out, as shown in Table 8, where the classification accuracy of the optimal feature combination of MOSPO-GDM is 0.0521, which is little different from 0.0531 in Table 5. This indicates that the combination of features optimized by the algorithm can indeed improve the recognition efficiency of AD patients. It also indirectly indicates the feasibility of the ten-fold cross strategy in this framework.

**Table 7:** The classification results (error rate: SVM, NB, and DA) of non-dominated solution using leave-one-out validation.

	**The classification error rate of non-dominated solution using leave-one-out validation**			
	**SVM**	**NB**	**DA**	**AVG**
NSGA	0.0388	0.1463	0.0815	0.0889
MOPSO	0.0159	0.1541	0.0828	0.0840
MOPSO-M	0.0116	0.1487	0.0819	0.0814
MOPSO-GDM	0.0130	0.1430	0.0808	0.0789

**Table 8:** The classification results (specific features and error rate: SVM, NB, and DA) of the optimal feature combination using leave-one-out validation.

	**The classification error rate of the optimal feature combination using leave-one-out validation**	
	**Feature**	**AVG error rate (SVM,NB,DA)**
NSGA	δ:EB-MD-MZ-HR,θ:AT,α:AT-MZ-HR,β:DG-SPL-GE,γ:DG-GIC	0.0624 (0.0122,0.1687,0.0061)
MOPSO	δ:MZ,θ: MZ,α: DG-AT-MD-MZ-HR,β:DG-GE-AT-MZ,γ: CC-SPL-EB-LE-MZ	0.0619 (0.0122,0.1478,0.0258)
MOPSO-M	δ:MZ,θ:MD,α:DG-AT-MZ-HR,β:DG-SPL-GE-AT-MZ,γ: DG-SPL-GE-MZ	0.0593 (0.0092,0.1533,0.0153)
MOPSO-GDM	δ:MZ,θ:AT,α:DG-AT-MZ-HR,γ:DG-MZ	0.0521 (0.0122,0.0981,0.0460)

Finally, several general feature selection algorithms with single-object optimization are used on the AD data in this paper, such as Recursive Feature Elimination (RFE), LASSO Regression, Feature selective validation (FSV), and Principal Component Analysis (PCA), the results are shown in Table 9, where C1, C2, and C2 respectively represent the classifier of SVM, NB, and DA. And in the PCA method, principal components are used as features. It can be seen that except for RFE, the classification results of the other three methods are poor. It is worth noting that the combination of features that worked well with SVM performed poorly with the other two classifiers, which shows that only relying on a single target or a single classifier to determine the combination of features is not universal. Therefore, multi-objective algorithm optimization is particularly significant.

**Table 9:** The results of general feature selection method: Recursive Feature Elimination (RFE), LASSO Regression, Feature selective validation (FSV) and Principal Component Analysis (PCA). C1: SVM; C2: NB; C3: DA analysis.

	**RFE**			**LASSO**			**FSV**			**PCA**		
**Feature Num**	**Classifier**			**Classifier**			**Classifier**			**Classifier**		
	**C1**	**C2**	**C3**	**C1**	**C2**	**C3**	**C1**	**C2**	**C3**	**C1**	**C2**	**C3**
2	0.0798	0.4722	0.5243	0.1748	0.2301	0.2117	0.2913	0.3465	0.2821	0.2452	0.2759	0.2544
3	0.1074	0.4691	0.3160	0.1871	0.2331	0.2178	0.1717	0.1810	0.1655	0.2483	0.2759	0.2544
4	0.0214	0.4507	0.1534	0.1410	0.2210	0.1656	0.1717	0.2024	0.1686	0.2544	0.2789	0.2544
5	0.0183	0.4477	0.1503	0.1379	0.2056	0.1441	0.1686	0.2085	0.1748	0.2544	0.2758	0.2482
6	0.0123	0.4416	0.1135	0.1379	0.1902	0.1379	0.1717	0.1963	0.1563	0.2574	0.2758	0.2513
7	0.0062	0.4416	0.1135	0.1379	0.1932	0.1348	0.1655	0.1901	0.1317	0.2574	0.2758	0.2696
8	0.0092	0.4355	0.1104	0.1318	0.1902	0.1256	0.1655	0.1993	0.1348	0.2544	0.2758	0.2635
9	0.0062	0.4294	0.1135	0.1349	0.1932	0.0735	0.1655	0.2023	0.1317	0.2544	0.2820	0.2698
10	0.0062	0.4110	0.1104	0.1409	0.1901	0.0918	0.1655	0.2177	0.1225	0.2544	0.2728	0.2698
12	0.0153	0.2638	0.1135	0.1409	0.1932	0.0888	0.1594	0.2208	0.1255	0.1286	0.2789	0.1410
14	0.0092	0.1410	0.1042	0.0368	0.1993	0.0765	0.1318	0.2085	0.1286	0.1317	0.2821	0.1471
16	0.0092	0.1349	0.1073	0.0428	0.1963	0.0642	0.1288	0.1963	0.1164	0.1132	0.2790	0.1380
18	0.0092	0.1349	0.1073	0.0306	0.1932	0.0612	0.1318	0.1902	0.1103	0.1132	0.2821	0.1349
20	0.0061	0.1349	0.1104	0.0306	0.1963	0.0703	0.1470	0.1932	0.1194	0.1163	0.2790	0.1134

## 6. Conclusion

In this work, the PSI method is used to quantify the synchronization strength of 16-channel EEG signals, leading to the construction of functional brain networks in AD patients. Analysis of the brain network in five frequency bands (δ, θ, α, β, and γ) shows that compared with the normal control group, the brain connections of AD patients reveal more sparsity. Then, fourteen topological features (DG-GIC) based on complex network formation patterns in EEG signals are extracted for Alzheimer's disease recognition. ANOVA statistical analysis and classification analysis are further applied to evaluate the effectiveness of topological features. Among which, most features significantly change in AD brain network when compared with the normal brain in the five frequency bands, which can effectively explain the disconnection characteristics of AD brain, resulting in the degeneration of AD brain function, especially in α, β, and γ frequency bands. Meanwhile, most of the features generated by SVM classifier have low classification error rates in the α, β, and γ frequency bands. Further, to enhance the classification performance, a novel hybrid and multi-objective optimization algorithm, MOPSO-GDM, is proposed for multiple feature combination (δ/ δ-θ-α-β-γ), achieving a much lower classification error rate than NSGA, MOPSO, and MOPSO-M algorithms, with a min classification error rate of 0.0531 (average error rate of SVM, NB, and DA classifiers) with 8 features combination in the δ-θ-α-β-γ. In sum, it is clear that applying the MOPSO-GDM for features selection can reduce the dimension of the feature set, which makes the resulting data set suitable for EEG-based AD clinical auxiliary diagnosis by minimizing the number of require features per sample to explore the potential markers and characterize the abnormalities of EEG signals of AD patients. Moreover, shedding light on EEG analysis of AD patients may help extend our understanding of brain function in patients with other neurological disorders. In future work, we will further explore the way features are fused with each other. Furthermore, such feature selection or dimensionality reduction methods as RFE and PCA give us new hints, which let us to consider the combination of intelligent optimization algorithms and non-genetic-feature selection methods to optimize AD brain network features in subsequent work.

## Data availability statement

The original contributions presented in the study are included in the article/Supplementary material, further inquiries can be directed to the corresponding author.

## Ethics statement

Written informed consent was obtained from the individual(s) for the publication of any potentially identifiable images or data included in this article.

## Author contributions

RW and HW designed the study and wrote the original draft. RW, HW, and LS provided the code support of algorithm. HW carried out the experiments. RW, HW, and QH analyzed the results. RW, HW, CH, YC, and LL polished the sentences of the article. RW, HW, QH, and YC modified the format of figures and tables. HW and QH finalize format of the manuscript. RW, CH, and YC provided the funding. All authors contributed to the article and approved the submitted version.
