# Supplementary material for: A novel framework of MOPSO-GDM in recognition of Alzheimer's EEG-based functional network
Source: Front Aging Neurosci. 2023 Jun 29;15:1160534. doi: 10.3389/fnagi.2023.1160534 (PMC10339813; doi:10.3389/fnagi.2023.1160534)
Supplement: Supplementary file 1 [file Data_Sheet_1.PDF]

Supplementary Material

1 SUPPLEMENTARY EXPLANATIONS OF TOPOLOGICAL FEATURES

1.1 Degree (DG)

Degree of node measures the number of links connected to a node. The greater the degree is, the more important the node is in the network (Rubinov and Sporns, 2010). Degree of a node i is defined by:

$$\mathrm{k_i} = \sum_{j \in N} a_{ij} \tag{S1}$$

1.2 Node Betweenness (NB)

Node betweenness is defined as the ratio of the number of paths passing through the node to the total number of shortest paths in the network (Kourtellis et al., 2013). It measures not only the importance of the node but also the amount of information flowing through the node k, as followed by:

$$B(k) = \sum_{i,j,k,i \neq j \neq k} \frac{d_{ij}(k)}{d_{ij}} \tag{S2}$$

where, $d_{ij}(k)$ is the shortest path between node i and j passing through the node k.

1.3 Clustering Coefficient (CC)

Clustering coefficient is an important parameter for measuring the degree of internal grouping and connection, reflecting the possibility of all neighboring nodes of a node being neighbors to each other. It describes the speed of information processing and transmission from the perspective of the network (Rubinov and Sporns, 2010; Watts and Strogatz, 1998). Clustering coefficient of the network is defined by:

$$C = \frac{1}{n} \sum_{i \in N} C_i = \frac{1}{n} \sum_{i \in N} \frac{2t_i}{k_i(k_i - 1)} \tag{S3}$$

where, C_i is the clustering coefficient of node i ($C_i = 0$ for $k_i < 2$).

1.4 Shortest Path Length (SPL)

Shortest path length assesses the influence of both local and distant functional connections. The shorter the path length, the stronger the functional integration, that is, the more direct connections between brain regions. Shortest path length between nodes i and j (Rubinov and Sporns, 2010) is defined by:

$$d_{ij} = \sum_{\mathrm{a}_{uv} \in g_{i \leftrightarrow j}} a_{uv} \tag{S4}$$

where, $g_{i \leftrightarrow j}$ is the shortest path between i and j. If node i and j are disconnected then $d_{ij} = \infty$.

1.5 Edge Betweenness (EB)

Edge betweenness is defined as the ratio of the number of paths passing through the edge to the total number of shortest paths in the network (Holme et al., 2002). It measures not only the importance of the edge but also the amount of information flowing through the edge.

$$B(k) = \sum_{<i,j>} \frac{d_{ij} < k, l >}{d_{ij}} \tag{S5}$$

where, $d_{ij} < k, l >$ is the shortest path passing through the edge $< k, l >$.

1.6 Global Efficiency (GE)

Global efficiency is a measure related to the average inverse shortest path length. As paths between disconnected nodes are defined to have infinite length, corresponding to zero efficiency, so the global efficiency may be meaningfully computed on disconnected networks, which make the global efficiency a superior measure of integration than the path length (Rubinov and Sporns, 2010; Latora and Marchiori, 2001). Global efficiency of the network is defined as:

$$E = \frac{1}{n} \sum_{i \in N} E_i = \frac{1}{n} \sum_{i \in N} \frac{\sum_{j \in N, j \neq i} d_{ij}^{-1}}{n-1} \tag{S6}$$

where, E_i is the efficiency of node i.

1.7 Local Efficiency (LE)

Local efficiency has the similar interpretation as clustering coefficient, but it can break down the limitation that only direct connections between neighbor nodes are taken into consideration in the calculation of clustering coefficient, so it can directly reflect the segregation index of a network (Rubinov and Sporns, 2010; Latora and Marchiori, 2001). Local efficiency of the network is defined as:

$$E_{loc} = \frac{1}{n} \sum_{i \in N} E_{loc,i} = \frac{1}{n} \sum_{i \in N} \frac{\sum_{j,h \in N, j \neq i} a_{ij} a_{ih} [djh(N_i)]^{-1}}{\mathrm{k}_i (k_i - 1)} \tag{S7}$$

where, $E_{loc,i}$ is the local efficiency of node i, and $djh(N_i)$ is the length of the shortest path between j and h, that contains only neighbors of i.

1.8 Transitivity (TT)

Transitivity refers to the probability that two adjacent nodes of the same node are still adjacent nodes, it is a classical variant of the clustering coefficient (Newman, 2003). Transitivity of the network is defined as:

$$T = \frac{\sum_{i \in N} 2ti}{\sum_{i \in N} ki(ki-1)} \tag{S8}$$

Note that transitivity is not defined for individual nodes.

1.9 Assortativity Coefficient (AC)

The assortativity coefficient is a correlation coefficient between the degrees of all nodes on two opposite ends of a link (Newman, 2002). The assortativity coefficient of a network can be positive or negative. Networks with a positive assortativity coefficient are therefore likely to have a comparatively resilient core of mutually interconnected high-degree hubs. On the other hand, networks with a negative assortativity coefficient are likely to have widely distributed and consequently vulnerable high-degree hubs. Assortativity coefficient of the network is defined as:

$$r = \frac{M^{-1}\sum_i j_i k_i - \left[M^{-1}\sum_i \frac{1}{2}\left(j_i + k_i\right)\right]^2}{M^{-1}\sum_i \frac{1}{2}\left(j_i^2 + k_i^2\right) - \left[M^{-1}\sum_i \frac{1}{2}\left(j_i + k_i\right)\right]^2} \tag{S9}$$

where, j_i, k_i are the degrees of the vertices at the ends of the ith edge, with $i = 1, 2..., M$.

1.10 Small-Worldness (SW)

Small-worldness is proposed to capture the high segregation and integration of small-world network (SWN). Thus it contains a integrated information of global and local network characteristics (Humphries and Gurney, 2008). A SWN is characterized by high local clustering and slightly higher global path length compared with the random network, thus in the SWNs, there is $S \gg 1$. Network small-worldness is defined as:

$$S = \frac{C/C_{rand}}{L/L_{rand}} \tag{S10}$$

where, C and C_rand are the clustering coefficients, and L and L_rand are the characteristic path lengths of the respective tested network and a random network.

1.11 Modularity (MD)

Newman (Newman, 2004) first proposed the modularity to quantify the degree to which the network may be subdivided into such clearly delineated and nonoverlapping groups. The modules connected by high-density nodes have few connections among different modules. The human brain has the characteristics of modularity, and each module undertakes different brain functions, which are called functional modules. supporting the functional segregation, which is the ability for specialized processing to occur within densely interconnected groups of brain regions. The modularity measure of a general network represents the maximum modularity of these modular methods. Although it can be seen from the clustering phenomenon of complex networks that the average clustering coefficient illustrates the modularity of networks to a certain extent, in the fact, modularity and clustering phenomenon are two different network characteristics (Rubinov and Sporns, 2010). Modularity of the network is defined as:

$$Q = \sum_{u \in M} \left[euu - \left(\sum_{v \in M} euv \right)^2 \right] \tag{S11}$$

the network is fully subdivided into a set of nonoverlapping modules M, and e_uv is the proportion of all links that connect nodes in module u with nodes in module v.

1.12 Motif Z-score (MZ)

Motifs are these patterns of local connectivity. motif z-score is the occurrence frequency of the motif, which is used to evaluate the significance of a motif in the network by comparison with ensembles of random null-hypothesis networks (Milo et al., 2002). Z-Score of motif is defined as:

$$z_h = \frac{J_h - \left\langle J_{rand,h} \right\rangle}{\sigma^{J_{rand,h}}} \tag{S12}$$

where, $\left\langle J_{rand,h} \right\rangle$ and $\sigma^{J_{rand,h}}$ are the respective mean and standard deviation for the number of occurrences of motif h in an ensemble of random networks.

1.13 Hierarchical Coefficient (HC)

Hierarchy is a fundamental characteristic of complex networks of modules. The hierarchy and the scale-free property impose strict restrictions on the number and the degree of cohesiveness of the different groups present in a network, which describes the dependence of the clustering coefficient on the node degree. The hierarchical coefficient can solve the problem that once we compute the distribution of the cluster coefficient according to connectivity $CC(k)$, the small world network reveals a plain distribution. All these networks follow the distinctive pattern (Ravasz and Barabási, 2003):

$$CC \sim k^{-\beta} \tag{S13}$$

where, the coefficient β is the hierarchical coefficient, and the equation represents the hierarchical property.

1.14 Graph Index Complexity (GIC)

By quantifying the complexity of two-dimensional data, Kim et al. introduced the graph index complexity as a new complex network topology parameter into the diagnosis of AD patients Kim and Wilhelm (2008). GIC is a description of the graphic complexity. It follows $0 \leq Cr \leq 1$. Suppose there is a graph with node number n and the maximum eigenvalue of its adjacency matrix is $\lambda_{\max}$, then GIC is defined as follows:

$$Cr = 4c_r\left(1 - c_r\right) \tag{S14}$$

where, all eigenvalues of the (symmetric) adjacency matrix of a graph G are real (Cvetkovic et al., 1980). The largest one is called index r of G. c_r can be expressed as:

$$c_r = \frac{r - 2\cos\left(\pi/\left(n+1\right)\right)}{n - 1 - 2\cos\left(\pi/\left(n+1\right)\right)} \tag{S15}$$

2 SUPPLEMENTARY TABLES

2.1 Tables

REFERENCES

Cvetkovic, D. M. et al. (1980). Spectra of graphs. theory and application. *PURE APPL. MATH.; USA; DA. 1980; VOL. 87; PP. 1-368; BIBL. 119 REF.*

Holme, P., Kim, B. J., Yoon, C. N., and Han, S. K. (2002). Attack vulnerability of complex networks. *Physical review E* 65, 056109. doi:10.1103/PhysRevE.65.056109

Table S1. One-way ANOVA results (F-Value and P-Value) of features in the δ, θ, α, β and γ frequency bands.

Band	δ		θ		α		β		γ	
Feature	F-value	P-value	F-value	P-value	F-value	P-value	F-value	P-value	F-value	P-value
DG	116.0423	**2.48E-23**	63.58686	**2.66E-14**	34.39619	**1.11E-08**	173.1147	**5.69E-32**	345.8748	**4.85E-53**
NB	103.1058	**3.25E-21**	82.73073	**9.72E-18**	51.45192	**5.02E-12**	196.5649	**3.13E-35**	155.6237	**1.95E-29**
CC	108.1921	**4.70E-22**	69.31428	**2.39E-15**	56.37166	**5.86E-13**	195.6256	**4.19E-35**	361.102	**1.26E-54**
SPL	82.14811	**1.23E-17**	38.31665	**1.82E-09**	20.82188	**7.16E-06**	222.2632	**1.23E-38**	149.5643	**1.55E-28**
EB	85.79047	**2.85E-18**	69.67825	**2.05E-15**	36.46863	**4.25E-09**	217.9183	**4.50E-38**	198.6849	**1.61E-35**
GE	101.2824	**6.55E-21**	50.39418	**7.99E-12**	27.78737	**2.48E-07**	193.6346	**7.84E-35**	347.9608	**2.93E-53**
LE	82.37323	**1.12E-17**	62.86833	**3.61E-14**	49.71766	**1.08E-11**	187.8696	**4.86E-34**	343.6343	**8.36E-53**
TS	122.4773	**2.31E-24**	49.84788	**1.02E-11**	27.19664	**3.28E-07**	158.1619	**8.26E-30**	286.1285	**1.90E-46**
AT	21.74515	**4.56E-06**	178.1298	**1.11E-32**	201.463	**6.81E-36**	160.4317	**3.84E-30**	10.27742	**0.001481**
SW	55.51238	**8.51E-13**	27.88219	**2.37E-07**	9.504643	**0.002226**	221.7154	**1.44E-38**	104.7788	**1.72E-21**
MD	118.1664	**1.13E-23**	40.20651	**7.67E-10**	14.80334	**0.000144**	220.7131	**1.95E-38**	270.0063	**1.48E-44**
MZ	87.38764	**1.50E-18**	0.028081	**0.867023**	40.90418	**5.58E-10**	129.4713	**1.83E-25**	0.239742	**0.624724**
HR	18.09709	**2.75E-05**	11.29289	**0.000871**	53.59206	**1.96E-12**	26.33559	**4.96E-07**	1.025078	**0.312073**
GIC	90.28006	**4.78E-19**	83.32935	**7.64E-18**	74.11783	**3.25E-16**	279.1036	**1.25E-45**	339.0914	**2.54E-52**

Humphries, M. D. and Gurney, K. (2008). Network 'small-world-ness': a quantitative method for determining canonical network equivalence. *PloS one* 3, e0002051. doi:10.1371/journal.pone.0002051

Kim, J. and Wilhelm, T. (2008). What is a complex graph? *Physica A: Statistical Mechanics and its Applications* 387, 2637–2652. doi:https://doi.org/10.1016/j.physa.2008.01.015

Kourtellis, N., Alahakoon, T., Simha, R., Iamnitchi, A., and Tripathi, R. (2013). Identifying high betweenness centrality nodes in large social networks. *Social Network Analysis and Mining* 3, 899–914. doi:10.1007/s13278-012-0076-6

Latora, V. and Marchiori, M. (2001). Efficient behavior of small-world networks. *Physical review letters* 87, 198701. doi:10.1103/PhysRevLett.87.198701

Milo, R., Shen-Orr, S., Itzkovitz, S., Kashtan, N., Chklovskii, D., and Alon, U. (2002). Network motifs: simple building blocks of complex networks. *Science* 298, 824–827. doi:10.1126/science.298.5594.824

Newman, M. E. (2002). Assortative mixing in networks. *Physical review letters* 89, 208701. doi:10.1103/PhysRevLett.89.208701

Newman, M. E. (2003). The structure and function of complex networks. *SIAM review* 45, 167–256. doi:10.1137/S003614450342480

Newman, M. E. (2004). Fast algorithm for detecting community structure in networks. *Physical review E* 69, 066133. doi:10.1103/PhysRevE.69.066133

Ravasz, E. and Barabási, A.-L. (2003). Hierarchical organization in complex networks. *Physical review E* 67, 026112. doi:10.1103/PhysRevE.67.026112

Rubinov, M. and Sporns, O. (2010). Complex network measures of brain connectivity: uses and interpretations. *Neuroimage* 52, 1059–1069. doi:10.1016/j.neuroimage.2009.10.003

Watts, D. J. and Strogatz, S. H. (1998). Collective dynamics of 'small-world'networks. *nature* 393, 440–442. doi:10.1038/30918
